# Supplementary material for: Antioxidant and Antimelanogenic Activities of Lactobacillus kunkeei NCHBL-003 Isolated from Honeybees
Source: Microorganisms. 2024 Jan 17;12(1):188. doi: 10.3390/microorganisms12010188 (PMC10818717; doi:10.3390/microorganisms12010188)
Supplement: Supplementary file 1 [file microorganisms-12-00188-s001.zip › microorganisms-2821749-supplementary.pdf]

Supplementary Table S1. Primer (human) sequences for real time PCR

Primer	Direction	Sequences (5' to 3')
HO-1	Forward	GGAGGAGATTGAGCGCAACA
	Reverse	CCAGACAGGTCACCCAGGTA
Nrf2	Forward	TGATTGACATACTTTGGAGGCAAGA
	Reverse	CGACTTCAACAGCAACTCCCACTCTTCC
GAPDH	Forward	CGACTTCAACAGCAACTCCCACTCTTCC
	Reverse	TGGGTGGTCCAGGGTTTCTTACTCCTT

Supplementary Table S2. Primer (mouse) sequences for real time PCR

Primer	Primer	Sequences
TYR	Forward	TGCACCTATCGGCCATAACA
	Reverse	ATACGACTGGCCTTGTTCCA
Mitf	Forward	TCTGAAGCAAGAGCATTGGC
	Reverse	CCGGATGTAGTCCACAGAGG
Tyrp-1	Forward	CTGGGAAAAACGTCTGCGAT
	Reverse	CTGATTGGTCCACCCTCAGT
Tyrp-2	Forward	TGGCTACAATTACGCCGTTG
	Reverse	CCCCAAGAGCAAGACGAAAG
GAPDH	Forward	AACTTTGGCATTGTGGAAGG
	Reverse	ACACATTGGGGGTAGGAACA
